# Supplementary material for: Ontogenetic Changes in the Feeding Behaviour of Helicoverpa armigera Larvae on Pigeonpea (Cajanus cajan) Flowers and Pods
Source: Plants (Basel). 2024 Feb 29;13(5):696. doi: 10.3390/plants13050696 (PMC10935201; doi:10.3390/plants13050696)
Supplement: Supplementary file 1 [file plants-13-00696-s001.zip › plants-2860041-supplementary.pdf]

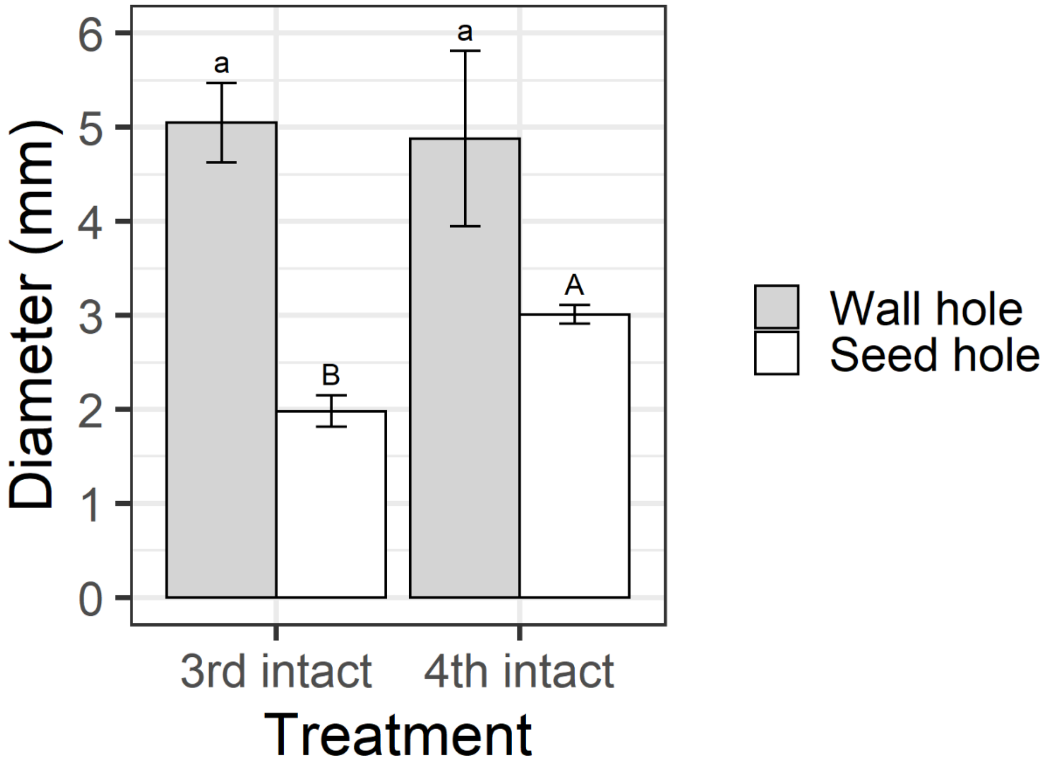

Figure S1. Mean hole diameter from the older instar pod feeding experiment. Only data from the intact pod treatment is presented/analysed, as larvae in the 'open' treatment had ready access to seeds. Wall holes indicates a larva has fed on the pod wall but not penetrated through to seed and seed holes indicate when the larva has penetrated through to the seed. Bars are the means and error bars are standard errors. Different letters indicate a significant difference between instars for the same hole type according to Fisher's LSD test.
